# Structural determinants of the integrin transmembrane domain required for bidirectional signal transmission across the cell membrane

**DOI:** 10.1016/j.jbc.2021.101318

**Published:** 2021-10-20

**Authors:** Zhengli Wang, Jieqing Zhu

**Affiliations:** 1 Blood Research Institute, Versiti, Milwaukee, Wisconsin, USA; 2 Department of Biochemistry, Medical College of Wisconsin, Milwaukee, Wisconsin, USA

**Keywords:** integrin, transmembrane domain, talin, cell adhesion, platelet, 2-BP, 2-bromopalmitate, BM, biotin-labeled maleimide, CT, cytoplasmic tail, MP, membrane-proximal, TM, transmembrane

## Abstract

Studying the tight activity regulation of platelet-specific integrin α_IIb_β_3_ is foundational and paramount to our understanding of integrin structure and activation. α_IIb_β_3_ is essential for the aggregation and adhesion function of platelets in hemostasis and thrombosis. Structural and mutagenesis studies have previously revealed the critical role of α_IIb_β_3_ transmembrane (TM) association in maintaining the inactive state. Gain-of-function TM mutations were identified and shown to destabilize the TM association leading to integrin activation. Studies using isolated TM peptides have suggested an altered membrane embedding of the β_3_ TM α-helix coupled with α_IIb_β_3_ activation. However, controversies remain as to whether and how the TM α-helices change their topologies in the context of full-length integrin in native cell membrane. In this study, we utilized proline scanning mutagenesis and cysteine scanning accessibility assays to analyze the structure and function correlation of the α_IIb_β_3_ TM domain. Our identification of loss-of-function proline mutations in the TM domain suggests the requirement of a continuous TM α-helical structure in transmitting activation signals bidirectionally across the cell membrane, characterized by the inside-out activation for ligand binding and the outside-in signaling for cell spreading. Similar results were found for α_L_β_2_ and α_5_β_1_ TM domains, suggesting a generalizable mechanism. We also detected a topology change of β_3_ TM α-helix within the cell membrane, but only under conditions of cell adhesion and the absence of α_IIb_ association. Our data demonstrate the importance of studying the structure and function of the integrin TM domain in the native cell membrane.

Integrins are a large family of cell surface receptors composed of α and β subunits. The combination of 18 α and eight β subunits in human forms 24 integrin heterodimers that interact with multiple extracellular or cell surface ligands and play diverse functions in different cell types (1). Both α and β subunits contain a large extracellular domain, a single-pass transmembrane (TM) domain, and usually a short cytoplasmic tail (CT). As a cell adhesion molecule, integrin can sense and transmit both chemical and mechanical signals, which are associated with local small-scale as well as long-range large-scale conformational changes (2, 3). A unique feature of integrin signaling is the bidirectional signal transmission across cell membrane, namely outside-in and inside-out signaling (1). The integrin TM domain not only acts as a membrane anchor but also plays a pivotal role in regulating the bidirectional signal transduction (4).

Current model of integrin activation regulation is largely based on the structural and functional studies of platelet-specific integrin α_IIb_β_3_ (5). The tight control of the on–off state of α_IIb_β_3_ is critical for the normal function of platelets in hemostasis. The resting α_IIb_β_3_ is maintained by a bent ectodomain structure involving extensive close contacts, which switches into an extended conformation upon activation (2, 5). Remarkably, the relatively simple and weak interactions at the TM and CT domains also contribute critically to the inactive state (4). The inside-out activation of α_IIb_β_3_ is triggered by the binding of intracellular proteins talin and kindlin to the β_3_ CT, leading to the disruption of α_IIb_β_3_ heterodimerization at the TM and CT domains (6). The destabilized α_IIb_β_3_ TM interaction then induces ectodomain extension on the cell surface, increasing affinity for binding with ligands such as fibrinogen in blood (5). Ligand binding to the ectodomain further induces conformational changes that are relayed to the CT through the TM domain, which induces the outside-in signaling for platelets adhesion, spreading, aggregation, and clot retraction (7). During this process, mechanical forces are generated by the cytoskeleton proteins binding to integrin CT and the extracellular ligands binding to integrin ectodomain. The integrin TM domain needs to resist the forces applied bidirectionally to the single-pass TM α-helical structure. How the conformational rearrangements of ectodomain are coupled with the structural changes of TM domain has been an active area of research.

Great efforts have been made in understanding the structural basis of the TM-CT domain in regulating α_IIb_β_3_ activation (8, 9, 10, 11, 12, 13). Although the heterodimeric structures of α_IIb_β_3_ TM-CT determined by different approaches share common structure features regarding the TM helix–helix packing (14), discrepancies remain about how the TM-CT association is maintained in an inactive state and whether and how the TM α-helix change its conformation upon activation (10, 11, 13, 15, 16, 17, 18). The β_3_ TM domain was often studied as a free isolated fragment in model membrane or detergent micelles, but inconsistent results were obtained regarding the oligomerization state and membrane topology (15, 18, 19, 20). In this study, we used proline scanning mutagenesis and cysteine accessibility by *in situ* biotin-maleimide labeling to analyze the structure of α_IIb_β_3_ TM domain in native cell membrane. Our data reveal the structural requirement of TM α-helix in regulating integrin bidirectional signal transduction, which also provides an example of how a rigid α-helical conformation participates in the signal transduction of single-pass cell membrane receptors.

## Results

### Effect of α_IIb_β_3_ transmembrane proline mutations on the ligand binding at resting condition

**Figure 1:**
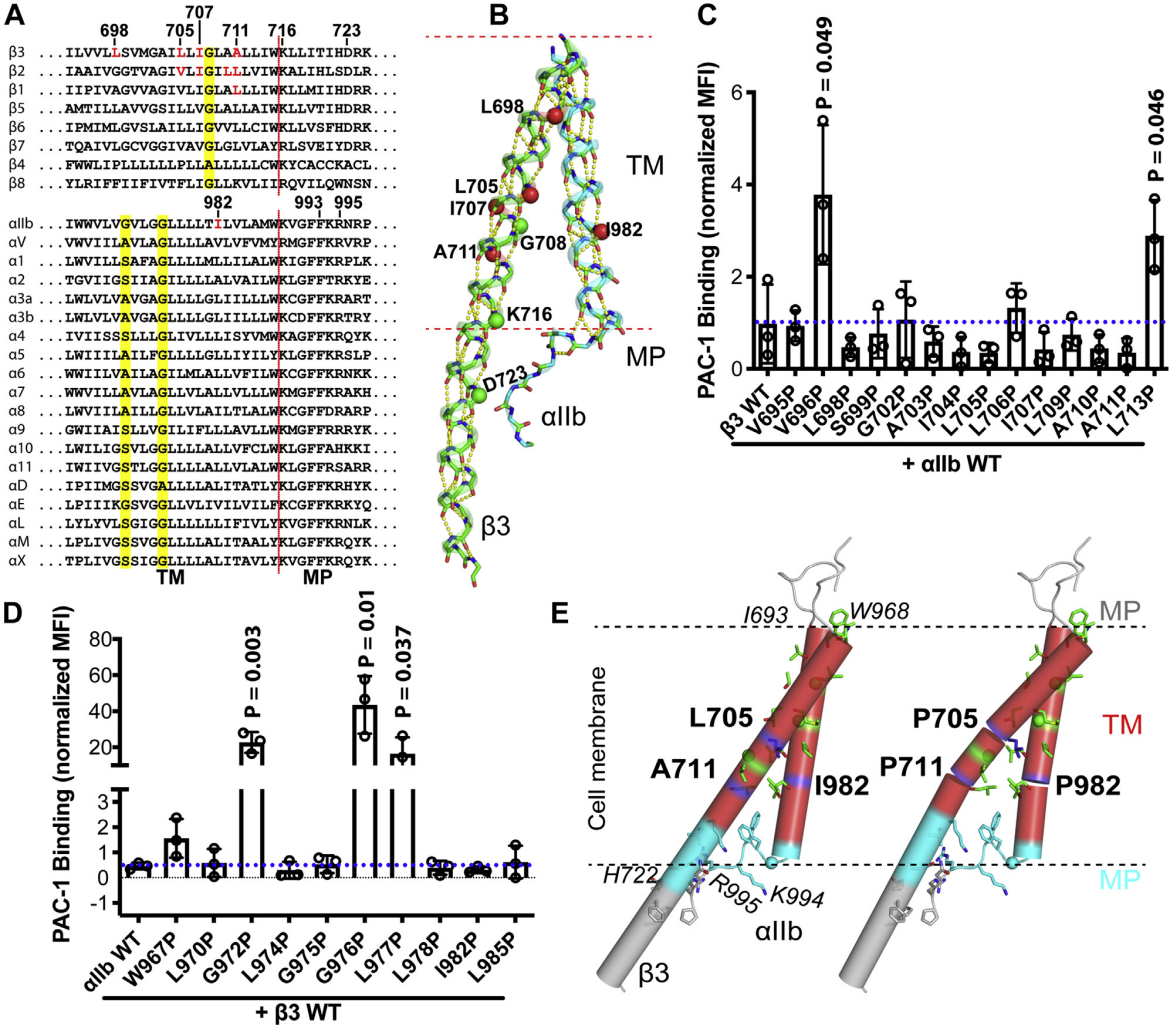
**Proline scanning of α**_**IIb**_**β**_**3**_**TM domains.***A*, sequence alignment of human integrin transmembrane domains. The predicted TM C-terminal boundary is marked with a *red dashed line*. The conserved small amino acids are highlighted in *yellow*. Shown in *red* are the residues that inhibit integrin inside-out activation when mutated to proline. *B*, backbone structure of α_IIb_β_3_ TM domain. Backbone hydrogen bonds are shown as *dashed lines* in *yellow*. Selected residues are marked as *spheres* of Cα atoms. *C* and *D*, ligand mimetic PAC-1 binding to α_IIb_β_3_ integrin with indicated proline substitutions. HEK293FT cells were transfected with indicated α_IIb_β_3_ constructs. The ligand-mimetic mAb PAC-1 binding was performed in the buffer containing 1 mM Ca^2+^/Mg^2+^ and detected by flow cytometry. The level of PAC-1 binding was normalized to α_IIb_β_3_ surface expression detected by mAb AP3 and shown as mean ± SD (n = 3). Unpaired two-tailed *t* test was performed between the control group without proline mutation and the group with proline mutation. Only *p* values less than 0.05 are shown. *E*, structural illustration of α_IIb_β_3_ TM domain within the cell membrane in the absence and presence of selected proline mutations. The proline mutations were introduced *in silico* to the TM structure using PyMOL. The proline-induced broken of a rigid α-helical structure was indicated. The interfacial residues are shown as *sticks* or Cα *spheres*. MP, membrane proximal.

Sequence alignment of the TM domains of 18 α and eight β human integrins shows typical TM features with hydrophobic residues that are rich in Leu, Ile, and Val (Fig. 1*A*). A conserved Gly residue (G708 in β_3_) in β integrins and two conserved small amino acids (Ala, Ser, or Gly) that form the GXXXG motif in α integrins (G972XXXG975 in α_IIb_) were identified to be critical in maintaining the resting state of α_IIb_β_3_ integrin (Fig. 1*A*) (21). Other conserved features that have been demonstrated to be important for the resting state include a conserved Lys at the TM inner boundary in both α and β integrins (11, 15), a conserved Asp in β membrane-proximal (MP) region (22), and a conserved GFFKR motif in the α MP region (23, 24) (Fig. 1*A*). Structure studies of α_IIb_β_3_ TM-CT heterodimer show a cross-angled straight α-helical structure for both α_IIb_ and β_3_ (10, 11, 13). The α-helical structure extends to the MP region in β_3_ subunit (Fig. 1*B*). A continued backbone hydrogen bonding network maintains the integrity of α-helical structure (Fig. 1*B*), which may be critical for the transmission of conformational signals across the cell membrane. To test this hypothesis, we performed proline scanning mutagenesis for α_IIb_β_3_ TM domain (Fig. 1, *C* and *D*), given that when present in an α-helical structure, proline tends to disturb the α-helical conformation by introducing a break or kink due to the lack of backbone hydrogen bonding (25) (Fig. 1*E*). Among the 14 proline mutations tested for β_3_ TM domain, most of them showed similar level of ligand-binding activity when measured with ligand-mimetic mAb PAC-1 under the resting condition (Fig. 1*C*). Several proline mutations, including β_3_-V696P and β_3_-L713P, and the previously identified β_3_-G708P and β_3_-K716P (11, 21), significantly rendered α_IIb_β_3_ more active than wild type (Fig. 1*C*). Among the ten proline mutations tested for α_IIb_ TM domain, seven of them showed a similar level of ligand binding as wild type under the resting condition (Fig. 1*D*) and three of them significantly rendered α_IIb_β_3_ constitutively active (Fig. 1*D*). All the active proline mutations, such as the β_3_-L706P and -G708P and the α_IIb_-G972P and -G976P, are located at or close to the α_IIb_β_3_ TM heterodimer interface, which may disturb the α_IIb_β_3_ TM interface leading to integrin activation.

### Identification of TM proline mutations that attenuate α_IIb_β_3_ inside-out activation

**Figure 2:**
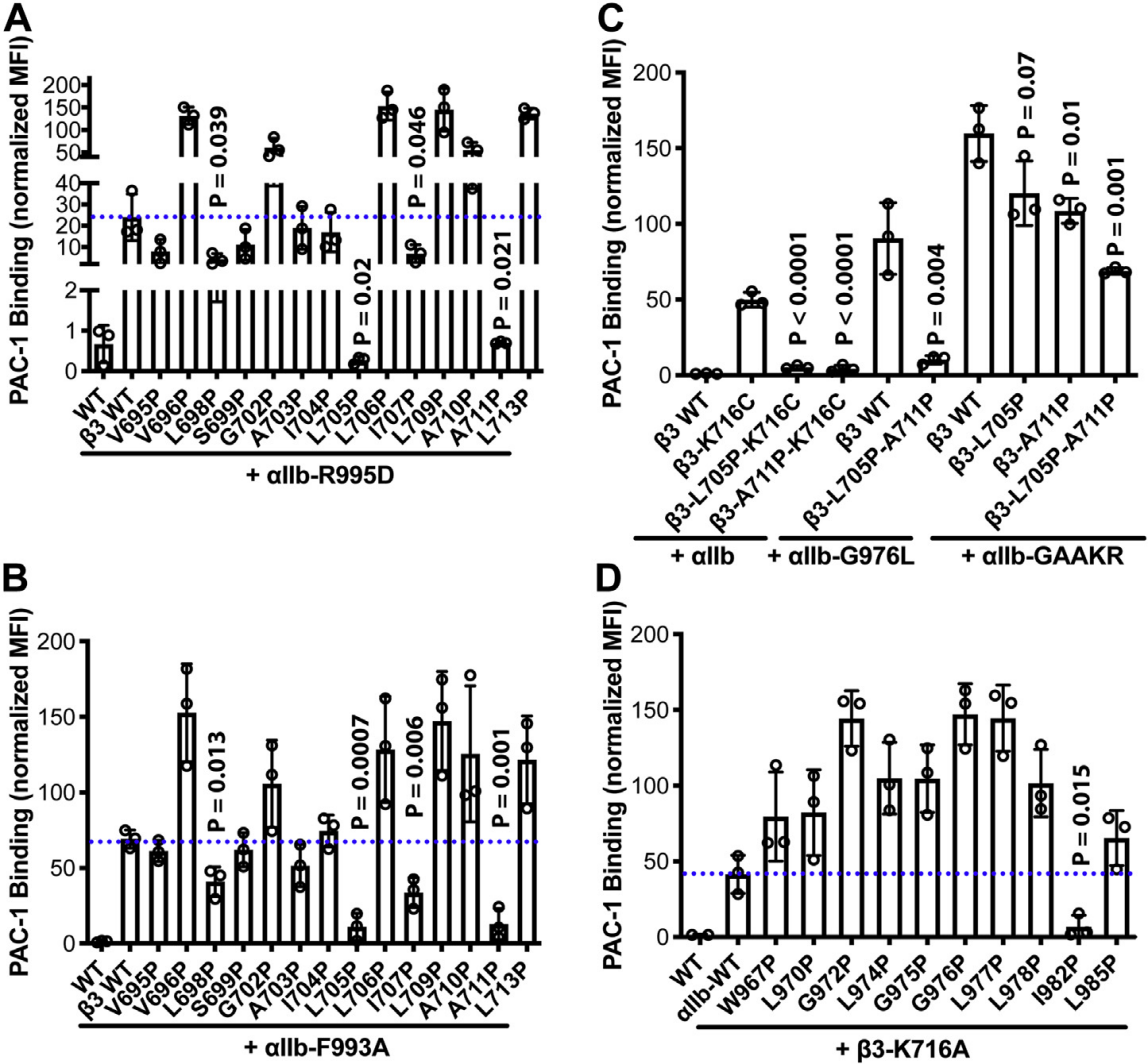
**Proline scanning of α**_**IIb**_**β**_**3**_**TM domains identified proline mutations that hindered activation.***A* and *B*, PAC-1 binding of β_3_ TM proline mutations on the background of the α_IIb_ cytoplasmic mutations, α_IIb_-R995D or α_IIb_-F993A that mimics inside-out activation of α_IIb_β_3_ integrin. *C*, PAC-1 binding of α_IIb_ TM proline mutations on the background of the β_3_ cytoplasmic mutations, β_3_-F993A that mimics inside-out activation of α_IIb_β_3_ integrin. *D*, PAC-1 binding of selected β_3_ TM proline mutations on the background of the α_IIb_ mutations, α_IIb_-G976L or α_IIb_-GAAKR that mimics inside-out activation of α_IIb_β_3_ integrin. HEK293FT cells were transfected with indicated α_IIb_β_3_ constructs. The PAC-1 binding was performed in the buffer containing 1 mM Ca^2+^/Mg^2+^ and detected by flow cytometry. The level of PAC-1 binding was normalized to α_IIb_β_3_ surface expression detected by mAb AP3 and shown as mean ± SD (n = 3). Unpaired two-tailed *t* test was performed between the control group without proline mutation and the group with proline mutation.

**Figure 3:**
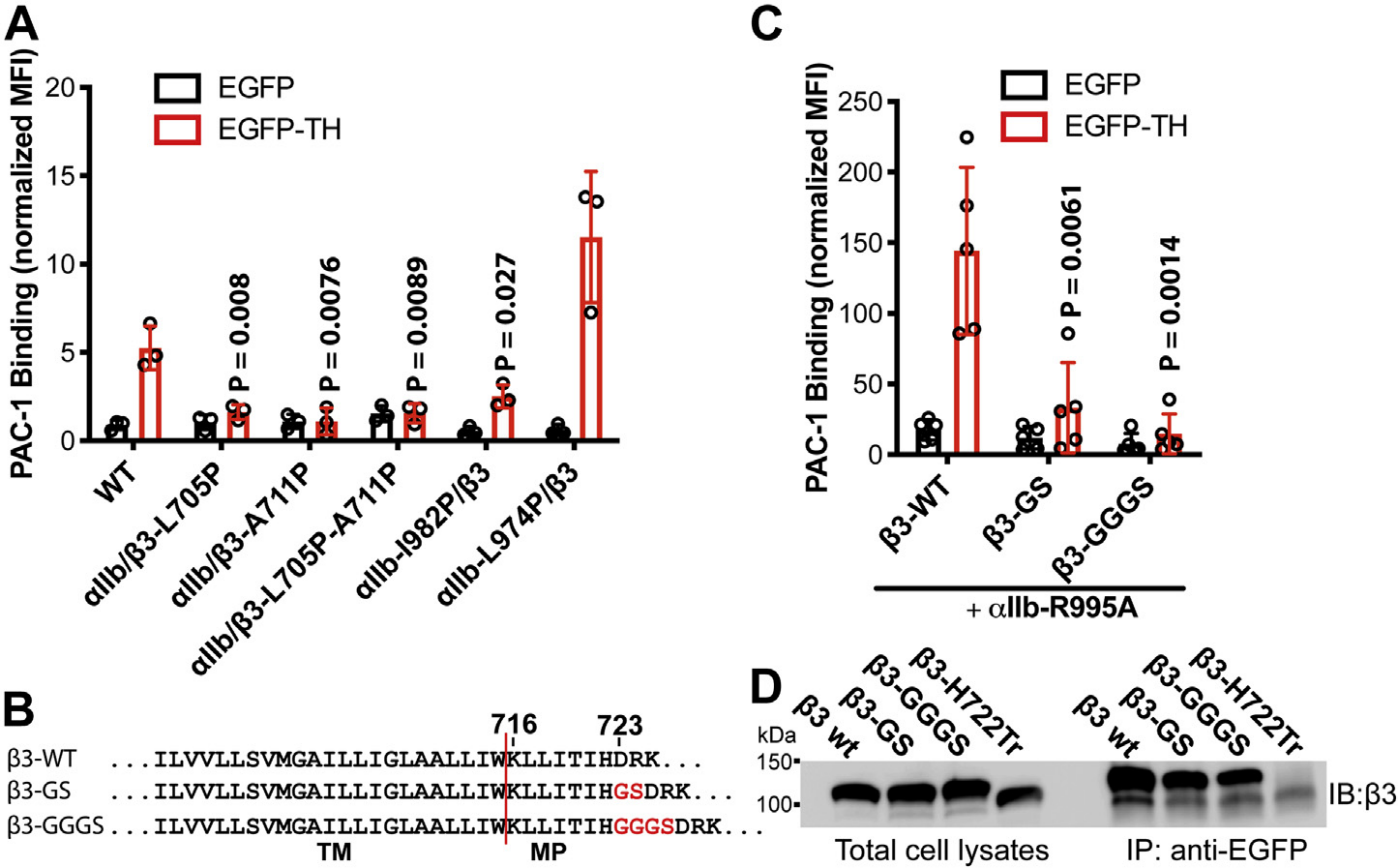
**Effect of disturbing the rigidity of TM and CT domains on talin1-head induced α**_**IIb**_**β**_**3**_**activation.***A*, effect of the selected α_IIb_β_3_ TM proline mutations on EGFP-talin-head (EGFP-TH) induced PAC-1 binding to α_IIb_β_3_ integrin. *B*, the β_3_-GS or β_3_-GGGS mutation was generated by the insertion of GS or GGGS sequence before the conserved β_3_-D723 residue. *C*, effect of β_3_-GS and β_3_-GGGS mutations on EGFP-TH induced PAC-1 binding to α_IIb_β_3_ integrin. HEK293FT cells were transfected with indicated α_IIb_β_3_ plus EGFP or EGFP-TH constructs. The PAC-1 binding was performed in the buffer containing 1 mM Ca^2+^/Mg^2+^ and detected by flow cytometry. The PAC-1 binding of EGFP and α_IIb_β_3_ double-positive cells was normalized to α_IIb_β_3_ surface expression detected by mAb AP3 and shown as mean ± SD (n ≥ 3). Unpaired two-tailed *t* test was performed between the control group without proline mutation and the group with proline mutation. *D*, interaction of β_3_-GS and β_3_-GGGS mutants with talin1-head domain. HEK293FT cells were transfected with α_IIb_ WT and indicated β_3_ plus EGFP-TH constructs. Total cell lysates were immunoprecipitated with anti-EGFP antibody and immunoblotted with anti-β_3_ antibody.

Having identified some TM proline mutations that reduced the basal level ligand binding of α_IIb_β_3_ under resting condition, we next tested the effect of these proline mutations on the inside-out activation of α_IIb_β_3_. Several activating mutations such as α_IIb_-R995D, α_IIb_-F993A, α_IIb_-F992A-F993A, and β_3_-K716A (or K716C), which are known to disturb the cytoplasmic domain association, have been widely used to mimic the inside-out activation of α_IIb_β_3_. When coexpressed with α_IIb_-R995D, four of the β_3_ TM proline mutations, including L698P, L705P, L707P, and A711P, significantly reversed the activating effect of α_IIb_-R995D (Fig. 2*A*), while the rest of proline mutations either had no effect or further enhanced the activation by α_IIb_-R995D (Fig. 2*A*). Similarly, when coexpressed with α_IIb_-F993A that is more activating than α_IIb_-R995D, the β_3_ L698P, L705P, I707P, and A711P significantly reversed the activating effect of α_IIb_-F993A (Fig. 2*B*). We next focused on β_3_ L705P and A711P for further analysis since their deactivating effect is more dramatic than others (Fig. 2, *A* and *B*). To test if the proline mutation also reverses the activating effect of β_3_ mutation, we generated L705P and A711P mutations in the context of β_3_-K716C. Both proline mutations significantly reduced the activation of α_IIb_β_3_ induced by β_3_-K716C (Fig. 3*C*). To test if the β_3_ L705P and A711P mutations have a synergistic effect, we coexpressed the single or double proline mutations with the highly active α_IIb_-F992A-F993A mutant. Both β_3_-L705P and β_3_-A711P reduced the activation by α_IIb_-F992A-F993A, while the β_3_-L705P-A711P double mutation further decreased the activation (Fig. 2*C*), demonstrating a synergistic effect. All the above activating mutations are expected to destabilize the α_IIb_β_3_ cytoplasmic interaction, which also disturbs TM association. We then tested a TM interface disturbing mutation α_IIb_-G976L. The β_3_-L705P-A711P also significantly reduced α_IIb_-G976L-mediated α_IIb_β_3_ activation (Fig. 2*C*). In contrast to β_3_ proline mutations, when coexpressed with the activating β_3_-K716A mutant, almost all the αIIb proline mutations further enhanced α_IIb_β_3_ activation, except α_IIb_-I982P that reduced the activation (Fig. 2*D*), indicating that the α_IIb_ TM domain tolerates proline mutation less than β_3_ TM domain.

### Effect of disturbing the rigidity of α-helical structures of TM and CT domains on talin1-head-induced α_IIb_β_3_ activation

Overexpression of the talin1 head domain is a well-established method to induce integrin inside-out activation (26). We used EGFP-tagged talin1 head (EGFP-TH) to induce α_IIb_β_3_ activation in HEK293FT cells (14). Compared with the EGFP control, EGFP-TH induced substantial α_IIb_β_3_ activation reported by PAC-1 binding (Fig. 3*A*). In contrast, the β_3_-L705P, β_3_-A711P, and β_3_-L705P-A711P mutations all completely abolished EGFP-TH-induced α_IIb_β_3_ activation (Fig. 3*A*). The α_IIb_-I982P mutation also significantly reduced EGFP-TH-induced α_IIb_β_3_ activation, while α_IIb_-L974P by contrast further enhanced α_IIb_β_3_ inside-out activation (Fig. 3*A*). The expression of EGFP-TH was comparable among all the transfections as determined by flow cytometry. These data are consistent with the above results obtained using the activating mutations that mimic α_IIb_β_3_ inside-out activation. Talin1 head induces α_IIb_β_3_ activation through binding to β_3_ cytoplasmic tail (CT) and disturbing α-β CT as well as TM associations (4). Our proline mutagenesis data suggest that the integration of TM α-helical structure is critical for talin1-head-induced conformational change of TM-CT domain. Since the α-helical structure of β_3_ TM extends to the cytoplasmic region (Fig. 1*B*), we asked if disturbing the integration of cytoplasmic α-helix also affects α_IIb_β_3_ activation. Instead of using proline mutagenesis, we inserted a flexible loop composed of GS or GGGS into the MP region of β_3_ CT (Fig. 3*B*). The insertion site is right before the conserved D723, given that the K716-H722 sequence is the minimal requirement for maintaining the resting state (14), and the sequence after H722 contains the talin1-binding sites (27). Compared with wild-type β_3_, both β_3_-GS and β_3_-GGGS mutants significantly reduced EGFP-TH-induced α_IIb_β_3_ activation (Fig. 3*C*). The longer GGGS insertion had more attenuated effect than the shorter GS insertion on α_IIb_β_3_ activation (Fig. 3*C*). The α_IIb_-R995A mutation was used to synergistically enhance the activation by EGFP-TH (14). The GS and GGGS mutations did not affect the binding of EGFP-TH to β_3_ CT as shown by the EGFP-TH pull-down assay (Fig. 3*D*). As a control, truncation of β_3_ CT after H722 (β_3_-H722Tr) completely abolished EGFP-TH binding (Fig. 3*D*). Altogether, these data suggest that the integrity of the α-helical structure at both the TM and CT domains of β_3_ is required for the inside-out activation of α_IIb_β_3_.

### TM proline mutations that attenuate the inside-out activation of β_2_ and β_1_ integrins

**Figure 4:**
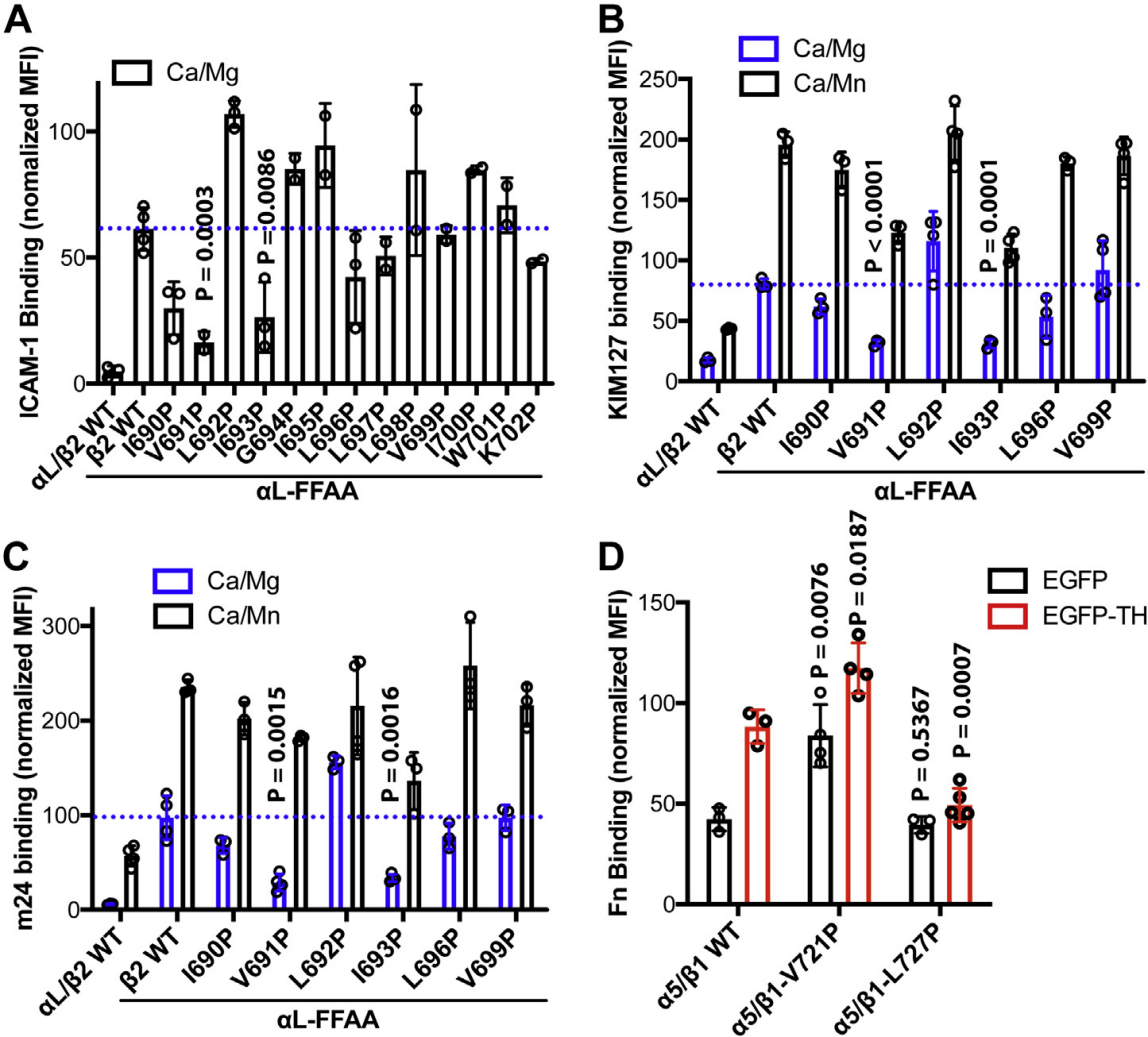
**Effect of TM proline mutations on the activation of β**_**2**_**and β**_**1**_**integrins.***A*, effect of TM proline mutations of β_2_ integrin on ICAM-1 binding to α_L_β_2_ integrin. The ICAM-1 binding to α_L_β_2_-transfected HEK293FT cells was performed in the presence of 1 mM CaCl_2_ plus 1 mM MgCl_2_ (Ca/Mg). *B* and *C*, effect of TM proline mutations of β_2_ integrin on the binding of activation-dependent mAbs, KIM127 and m24. The mAb binding to α_L_β_2_-transfected HEK293FT cells was performed in the presence of 1 mM Ca/Mg or 1 mM CaCl_2_ plus 1 mM MnCl_2_ (Ca/Mn). *D*, effect of TM proline mutations of β_1_ integrin on talin-head (TH)-induced fibronectin (Fn) binding to α_5_β_1_ integrin. α_5_β_1_-KO HEK293FT cells were transfected with α_5_β_1_ plus EGFP or EGFP-TH constructs. The binding of Alexa Fluor 647-labled human fibronectin (Fn) was performed in the presence of 1 mM Ca/Mg. Both the ligand and the LIBS mAb binding were normalized to integrin expression. Data are mean ± SD (n ≥ 3). Unpaired two-tailed *t* test was performed between the control group without proline mutation and the group with proline mutation.

We expanded our studies to β_2_ integrin to ask if certain TM proline mutations also affect the inside-out activation of α_L_β_2_ integrin. We screened 13 proline mutations for the C-terminal half of the β_2_ TM domain, which includes the residues β_2_-V691 and -L697 that are equivalent to β_3_-L705 and -A711, respectively (Figs. 1*A* and 4*A*). Using the α_L_-GFFKR to GAAKR mutation to induce inside-out α_L_β_2_ activation reported by soluble ICAM-1 binding, we found that six β_2_ TM proline mutations reduced α_L_β_2_ activation (Fig. 4*A*). Most of the inhibitory proline mutations of β_2_ are equivalent to those found in β_3_ integrin, such as β_2_-I690P, -V691P, and -I693P that are equivalent to β_3_-I704P, -L705P, and -I707P, respectively (Fig. 1*A*). Similar to β_3_-L705P, the equivalent β_2_-V691P has much profound negative effect. However, the β_3_-A711P equivalent mutation, β_2_-L697P, only slightly reduced α_L_β_2_ activation (Fig. 4*A*). To correlate the effect of proline mutations on ligand binding with large-scale conformational changes of ectodomain, we used two mAbs KIM127 and m24 to report β_2_ extension and headpiece open, respectively (28, 29). Consistent with the ICAM-1 binding results, β_2_-V691P and -I693P mutations significantly reduced the α_L_-GAAKR-induced binding of KIM127 and m24 (Fig. 4, *B* and *C*), suggesting that the TM proline mutations restrained the conformational activation signal transmitted across the cell membrane. In contrast, Mn^2+^, an integrin activator for the extracellular domain, still stimulates KIM127 and m24 binding in the presence of the inhibitory proline mutations (Fig. 4, *B* and *C*). We also tested the β_3_-L705P and -A711P equivalent mutations on β_1_ integrin, namely β_1_-V721P and -L727P. The fibronectin-binding assay showed that the β_1_-L727P mutation significantly reduced while the β_1_-V721P significantly enhanced EGFP-TH-induced α_5_β_1_ activation (Fig. 4*D*). These data suggest that the integrity of TM α-helical structure is also important for the inside-out activation of β_1_ and β_2_ integrins.

### The β_3_-L705P-A711P mutation dampens α_IIb_β_3_-mediated outside-in signaling

**Figure 5:**
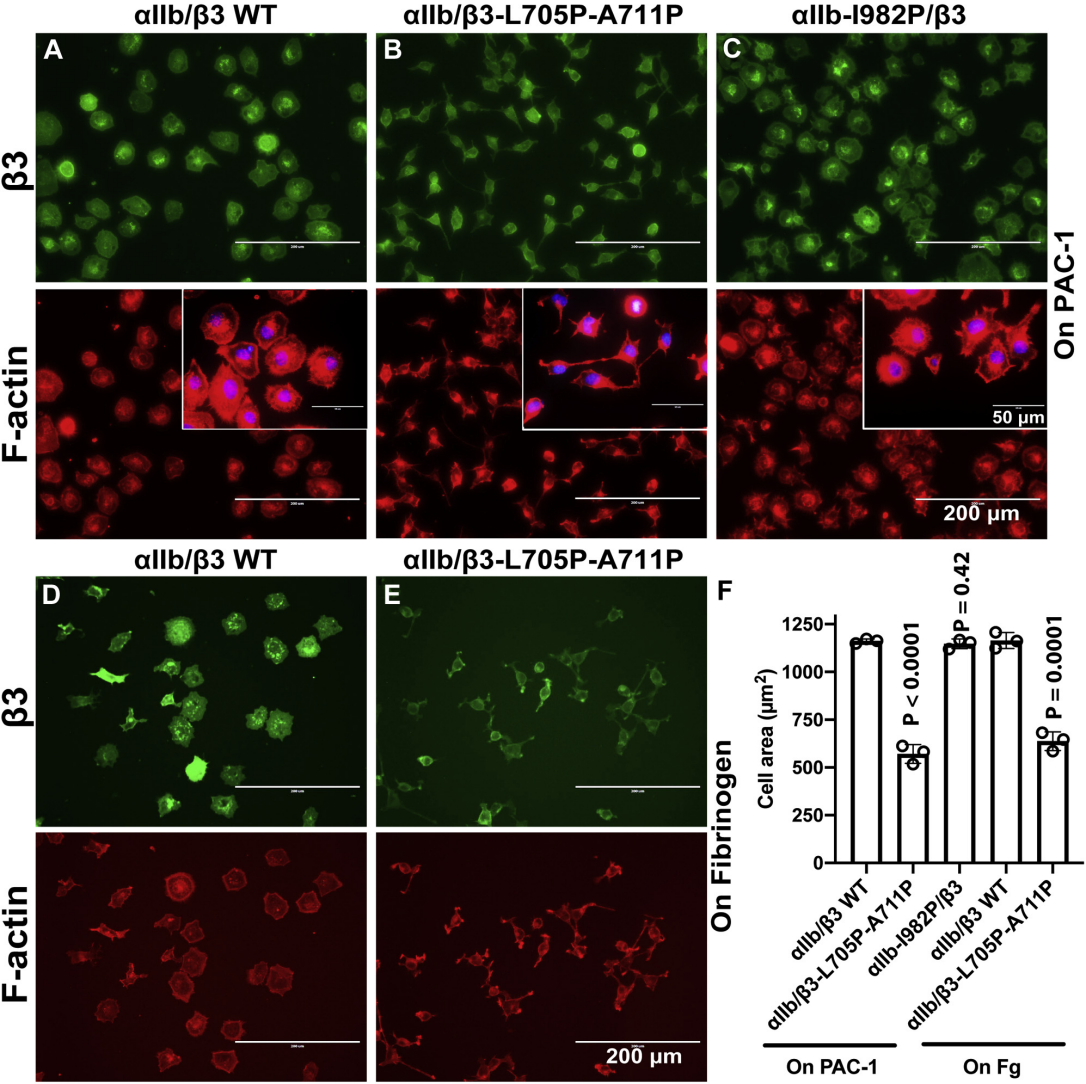
**Effect of TM proline mutations on α**_**IIb**_**β**_**3**_**-mediated cell spreading.***A*–*C*, HEK293 cells stably expressing α_IIb_/β_3_ WT, α_IIb_/β_3_-L705P-A711P, or α_IIb_-I982P/β_3_ spread on immobilized PAC-1. *D* and *E*, HEK293 cells stably expressing α_IIb_/β_3_ WT or α_IIb_/β_3_-L705P-A711P spread on immobilized fibrinogen. Cells were allowed to adhere on platelets coated with 5 μg/ml PAC-1 or 25 μg/ml fibrinogen at 37 °C for 1 h and then fixed and stained with Alexa-Fluor-488-labeled AP3 for β_3_ detection and Alexa-Fluor-564-labeled phalloidin for F-actin detection. Nuclei were stained with DAPI in panels *A*–*C*. *F*, quantification of cell spreading areas in panels *A*–*E*. The averaged cell areas were calculated based on 30 to 50 cells for each of three independent repeats. Data are mean ± SD. Unpaired two-tailed *t* test was performed between the WT and the mutant cells.

Integrin conformational signal is transmitted bidirectionally across the TM domain. Having found that the β_3_-L705P-A711P mutation greatly reduced the inside-out activation of α_IIb_β_3_ integrin, we further asked whether such mutation also affects α_IIb_β_3_ outside-in signaling, in which extracellular ligand binding induces large-scale conformational changes that are transmitted to the CT through the TM α-helix (2). We generated HEK293 cell lines stably expressing comparable levels of α_IIb_β_3_ wild type, α_IIb_/β_3_-L705P-A711P, or α_IIb_-I982P/β_3_. The α_IIb_β_3_-mediated cell spreading on the immobilized ligand, a hallmark of integrin outside-in signaling, was comparably measured among the stable cell lines. When seeded on the plate coated with ligand-mimetic mAb PAC-1, the HEK293-α_IIb_β_3_-WT cells showed substantial cell spreading (Fig. 5*A*). By contrast, the HEK293-α_IIb_/β_3_-L705P-A711P cells had dramatic defects of cell spreading, showing much smaller spreading area than the WT cells (Fig. 5, *B* and *F*), although there were no obvious differences in the number of attached cells. Interestingly, the HEK293-α_IIb_-I982P/β_3_ cells spread as well as the WT (Fig. 5, *C* and *F*). Consistently, the HEK293-α_IIb_/β_3_-L705P-A711P cells also showed defective cell spreading on the physiological ligand fibrinogen (Fig. 5, *D*–*F*). These data suggest that the integrity of the α-helical structure of β_3_ TM domain is required for the outside-in signaling.

### High-affinity soluble ligand binding does not induce conformational changes of α_IIb_β_3_ transmembrane domain detected by biotin-maleimide (BM) labeling

**Figure 6:**
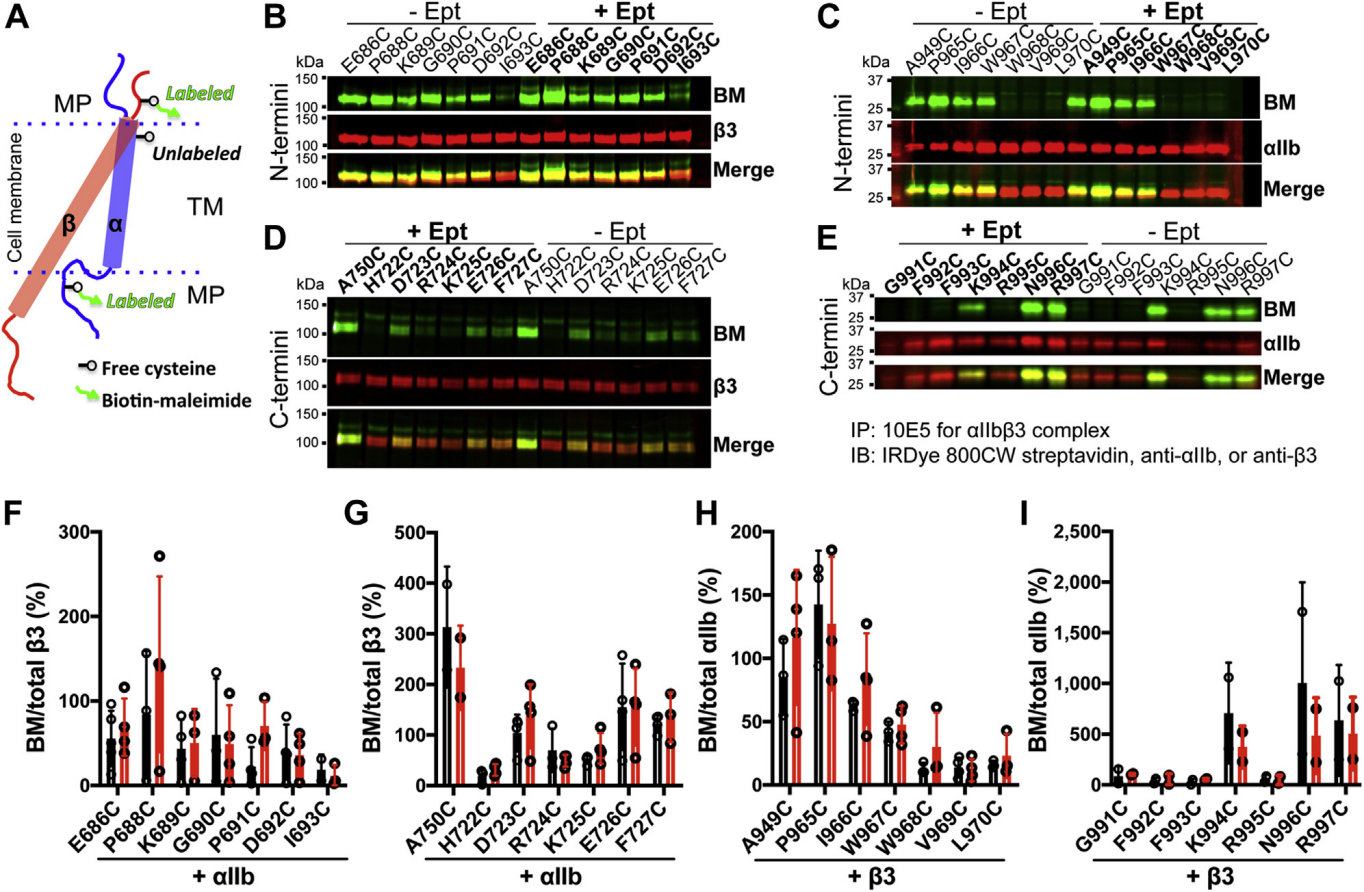
**BM-labeling of α**_**IIb**_**β**_**3**_**TM cysteine substitutions before and after soluble ligand binding.***A*, the cysteine scanning accessibility method for determining the burial/exposure status of integrin TM domain. A single cysteine mutation was introduced into the α or β TM domain. The cysteine located outside of the cell membrane can be labeled by biotin-maleimide (BM), while the cysteine buried in the cell membrane is not accessible to the labeling. *B*–*E*, the BM labeling of substituted cysteines in the β_3_ (*B* and *D*) and α_IIb_ (*C* and *E*) TM domains. HEK293FT cells were transfected with wild-type α_IIb_ plus β_3_ containing a single-cysteine mutation or wild-type β_3_ plus α_IIb_ containing a single-cysteine mutation. The cells were treated with or without the high-affinity ligand eptifibatide (Ept) and labeled with BM on ice. The α_IIb_β_3_ was immunoprecipitated with α_IIb_β_3_-complex specific mAb 10E5 and immunoblotted with indicated detection reagents. *F*–*I*, quantification of western blot data as described in panels *B*–*E*. Data are mean ± SD (n ≥ 2).

Having found that the rigid α-helical structure of TM domain is critical for the bidirectional integrin activation, we next asked if the α-helix performs conformational change within the cell membrane. Such conformational changes may be disrupted due to the helix-breaking effect of a proline mutation (Fig. 1*E*). To measure the potential conformational change of TM α-helix in the cell membrane, we performed the cysteine scanning accessibility assay (30). The membrane-permeable sulfhydryl-specific reagent, biotin-labeled maleimide (BM), was used to label the substituted cysteine residues. Since the reaction only occurs in an aqueous environment, the cysteines residing in the lipid bilayer cannot be labeled (Fig. 6*A*), which will show the burial/exposure status of TM residues. By comparing the resting and active states, the changes of labeling accessibility of the substituted cysteines indicate the changes of membrane embedding of TM α-helix. We first tested whether ligand-induced large-scale conformational change at the ECD induces structural rearrangement of TM domains. The RGD-mimetic high-affinity drug eptifibatide was used to induce the headpiece opening and extension of α_IIb_β_3_ (31, 32). Both the N-terminal and C-terminal membrane-proximal cysteine mutations of α_IIb_β_3_ TM domains were analyzed by BM labeling in the absence or presence of eptifibatide. 2-Bromopalmitate (2-BP) was used to inhibit the potential palmitoylation of C-terminal cysteines (11). The signals of BM labeling and total protein were simultaneously detected by western blot after immunoprecipitation (Fig. 6, *B*–*E*). BM labeling of α_IIb_β_3_ TM N-terminal cysteine mutations showed that the β_3_-I693C and α_IIb_-W697C are the last residues giving detectable BM signal (Fig. 6, *B* and *C*), suggesting they are the N-terminal boundary residues of α_IIb_β_3_ TM domains. BM labeling of β_3_ TM C-terminal cysteine mutations suggested that the C-terminal TM boundary of β_3_ is at the residues I721H722 (Fig. 6*D*). The BM labeling of α_IIb_ TM C-terminus showed an interesting pattern, suggesting that the residues G991C, F992C, F993C, and R995C are all embedded in the cell membrane, while K994C and the residues after R995 are exposed (Fig. 6*E*). This data is consistent with the loop conformation of α_IIb_ GFFKR motif as determined by structural studies (11, 13) (Fig. 1, *B* and *E*). Compared with the results without eptifibatide, eptifibatide did not induce obvious changes in the BM labeling patterns for both α_IIb_ and β_3_ subunits, suggesting no changes in membrane embedding (Fig. 6, *B*–*I*).

### BM labeling of α_IIb_ cytoplasmic membrane-proximal region reveals no obvious conformational changes related to activation

**Figure 7:**
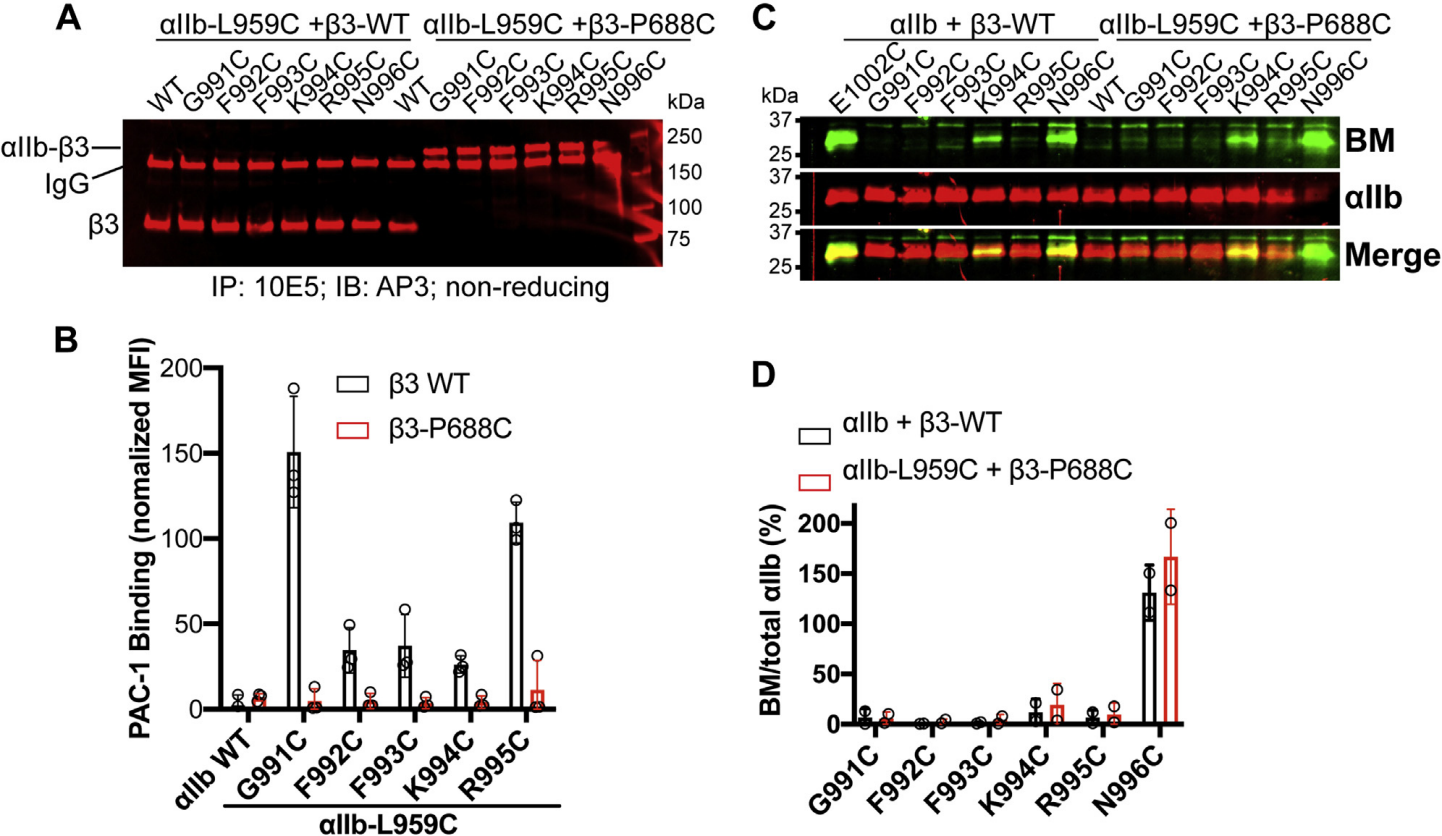
**BM labeling of the cysteine substitutions in the membrane-proximal (MP) region of α**_**IIb**_**cytoplasmic tail.***A*, the introduction of a disulfide bond that cross-links the α_IIb_ and β_3_ subunit at the N-terminus of TM domain. HEK293FT cells were transfected with β_3_ WT or β_3_-P688C mutant and the α_IIb_ MP cysteine mutants in the presence of L959C mutation. The α_IIb_β_3_ proteins were immunoprecipitated with mAb 10E5 and subjected to immunoblot with anti-β_3_ mAb AP3 under nonreduced condition. *B*, disulfide bond formation between α_IIb_-L959C and β_3_-P688C reversed the α_IIb_β_3_ activation induced by the cysteine substitutions at the α_IIb_ MP region. HEK293FT cells were transfected with indicated α_IIb_ constructs plus β_3_-WT or β_3_-P688C mutant. PAC-1 binding to the transfected cells was performed in the presence of 1 mM Ca/Mg. Data are mean ± SD (n ≥ 2). *C*, BM labeling of α_IIb_ MP cysteine mutants in the absence and presence of the disulfide bond formed between α_IIb_-L959C and β_3_-P688C. *D*, quantification of western blot data as described in panel *C*. Data are mean ± SD (n ≥ 2).

Previous studies showed that the cysteine mutations of α_IIb_ cytoplasmic GFFKR motif rendered α_IIb_β_3_ constitutively active (11), suggesting that the BM labeling pattern of GFFKR cysteine mutations might be related to the active conformation (17). To reverse the activating status of GFFKR cysteine mutations, we introduced additional α_IIb_-L959C and β_3_-P688C mutations at the N-terminal MP region of TM domain, which was known to form an interchain disulfide bridge that restrains the α/β TM separation (33). Western blot under nonreducing conditions demonstrated 100% disulfide bond formation for all the mutants tested (Fig. 7*A*). Ligand-mimetic PAC-1 binding showed that α_IIb_-L959C/β_3_-P688C mutations reversed the activating effect of the GFFKR cysteine mutations (Fig. 7*B*). However, there are no obvious changes in the BM labeling for the GFFKR motif in the presence of α_IIb_-L959C-β_3_-P688C disulfide bond (Fig. 7, *C* and *D*), suggesting that the activating cysteine mutations do not change the membrane embedding of the α_IIb_ GFFKR motif. Thus, the membrane embedding of GFFPR revealed by BM labeling should represent the resting state.

### BM labeling of β_3_ TM N-terminal membrane-proximal region under cell adhesion or overexpression of EGFP-TH

**Figure 8:**
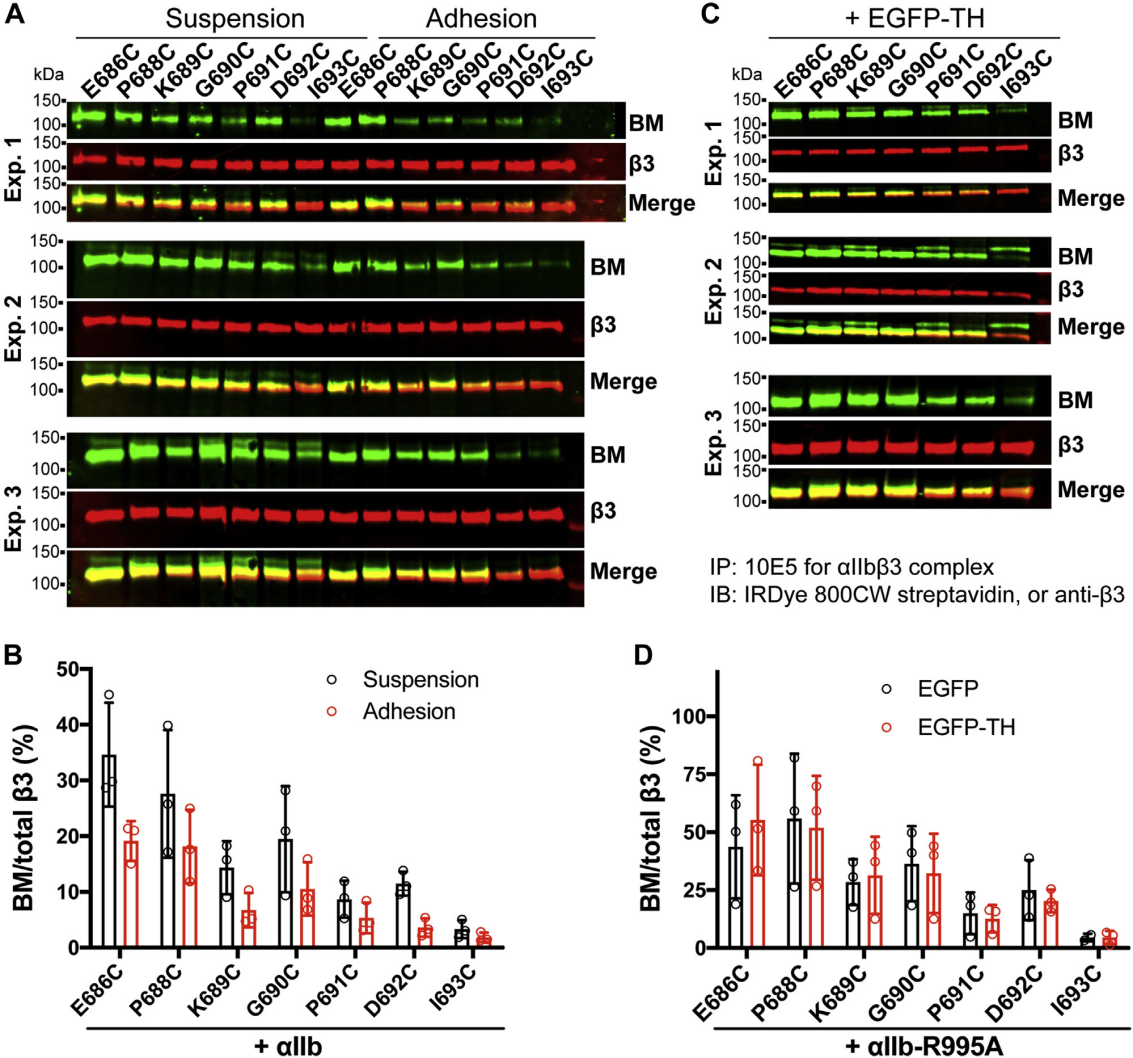
**BM labeling of the cysteine substitutions in the β**_**3**_**TM domain upon cell adhesion or EGFP-TH-induced α**_**IIb**_**β**_**3**_**activation.***A*, HEK293FT cells were transfected with α_IIb_ WT plus indicated β_3_ cysteine mutants. The transfected cells were kept in suspension or allowed to adhere on fibrinogen-coated surface and labeled with BM. *B*, quantification of western blot data of panel *A*. *C*, HEK293FT cells were transfected with EGFP-TH and α_IIb_-R995A plus indicated β_3_ cysteine mutants. The cells were labeled with BM in suspension. *D*, quantification of western blot data of panel *C*. Data are mean ± SD of three independent experiments.

During integrin-mediated cell adhesion and spreading, in addition to the ligand-induced long-range conformational changes that are transmitted to the CT through TM domain, the tensile force generated by ectodomain interacting with immobilized extracellular ligand and CT interacting with intracellular cytoskeleton is also applied to TM domain (34), which may affect the membrane embedding of TM α-helix. We performed the BM labeling of the N-terminal MP region of β_3_ TM domain for the cells spreading on immobilized fibrinogen. Compared with the BM labeling of suspension cells, we consistently observed a decrease of BM labeling for the residues of β_3_-K689C, β_3_-P691C, β_3_-D692C, and β_3_-I693C that are at the β_3_ TM outer boundary (Fig. 8, *A* and *B*), suggesting a change of membrane embedding of β_3_ TM α-helix in the spreading cells. Talin1 head has been reported to induce conformational change of β_3_ TM domain (35). We also did BM labeling of β_3_ TM N-terminal cysteine mutants in the presence of overexpression of EGFP-TH. α_IIb_-R995A mutant was used to synergize the activating effect of EGFP-TH (14). EGFP-TH induced comparable activation of all the tested β_3_ cysteine mutants (data not shown). However, compared with the cells with EGFP expression, no obvious changes of BM labeling were detected for the N-terminal MP residues of β_3_ TM α-helix (Fig. 8, *C* and *D*). These data suggest that mechanical force but not talin1 head may induce a change of β_3_ membrane embedding.

### BM labeling of free β_3_ TM domain in the absence of α_IIb_ subunit

**Figure 9:**
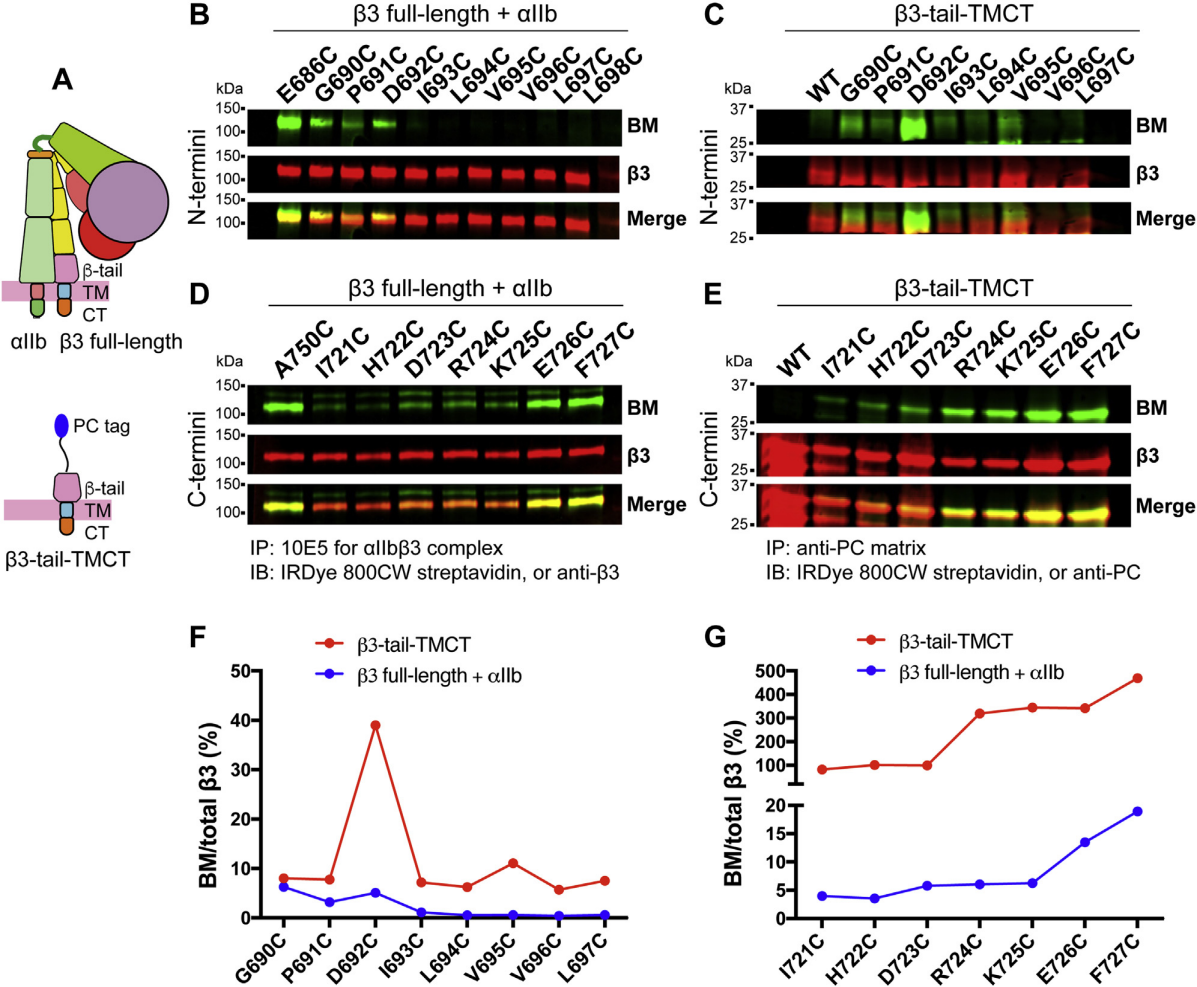
**BM labeling of the cysteine substitutions in the β**_**3**_**TM domain that was fully separated from the α**_**IIb**_**subunit.***A*, design of β_3_-tail-TMCT construct. A protein C tag was added to the N-terminus of β_3_-tail-TMCT construct. *B* and *D*, BM labeling of the cysteine substitutions in the TM domain of full-length β_3_ coexpressed with α_IIb_ in HEK293FT cells. *C* and *E*, BM labeling of the cysteine substitutions in the TM domain of β_3_-tail-TMCT expressed in HEK293FT cells. *F*, quantification of western blot data of panels *B* and *C*. *G*, quantification of western blot data of panels *D* and *E*. Data are presented as BM signal as a percentage of total β_3_ signal.

The current model suggests a separation of α and β TM domains upon integrin activation (2). The β_3_ TM α-helix has been studied as an isolated single-chain peptide (15, 20), which may represent an active form separated from α_IIb_ TM association. To investigate the membrane embedding of β_3_ TM α-helix by BM labeling in the absence of α_IIb_ subunit, we generated a β_3_ construct containing the β-tail, TM, and CT domains with an N-terminal protein C (PC) tag, namely β_3_-tail-TMCT (Fig. 9*A*). The β_3_-tail-TMCT construct could be expressed on the cell surface without α_IIb_ subunit as determined by flow cytometry (data not shown), suggesting that it may mimic a free state of β_3_ TM domain that is fully separated from α_IIb_ subunit. Both the N- and C-terminal MP regions of β_3_ TM domain were comparably studied by BM labeling for β_3_-tail-TMCT and β_3_ full-length constructs (Fig. 9, *B*–*E*). BM labeling of β_3_ full-length cysteine mutants coexpressed with α_IIb_ subunit shows that the N-terminal boundary of β_3_ TM domain is at residue β_3_-I693 (Fig. 9*B*). In contrast, the elevated BM labeling of β_3_-tail-TMCT suggested the changes of TM boundary and membrane embedding (Fig. 9, *C* and *F*). Consistently, compared with the β_3_ full-length, there was an obvious increase of BM labeling for the C-terminal residues, such as β_3_-R724C and β_3_-K725C in β_3_-tail-TMCT (Fig. 9, *D*, *E*, and *G*), suggesting a change of β_3_ membrane embedding. Our BM labeling data for the full-length β_3_ complexed with α_IIb_ indicate that the β_3_ TM α-helix needs to be tilted in the lipid bilayer to accommodate membrane embedding (Fig. 1*E*). Upon disassociation from the α_IIb_ TM domain, the β_3_ TM α-helix may adopt a different tilt within the cell membrane as shown by our BM labeling analysis.

## Discussion

Mutagenesis studies have identified numerous activating mutations at the α_IIb_β_3_ TM domains (8, 11, 21), which were known to destabilize the α_IIb_β_3_ TM association. In this study, we used proline scanning mutagenesis to identify TM proline mutations that either reduce or enhance α_IIb_β_3_ inside-out activation. Proline residues present in an α-helix tend to disrupt the integrity of the rigid α-helical structure by introducing a kink due to the interruption of backbone hydrogen bonds (25). As a result, it is expected that some proline mutations may disturb the TM association and render α_IIb_β_3_ more active than wild type. However, it is remarkable that proline mutations at certain positions also greatly reduced α_IIb_β_3_ inside-out activation triggered by either activating mutations or EGFP-TH. Such proline mutations are distributed at the N-terminus (β_3_-L698P), middle (β_3_-L705P and -I707P), and C-terminus (β_3_-A711P) of β_3_ TM domain (Fig. 1*A*), which displayed different levels of inhibitory effect on α_IIb_β_3_ inside-out activation (Fig. 2*B*). Similarly, the β_3_ equivalent TM proline mutations also inhibited the inside-out activation of α_L_β_2_ and α_5_β_1_ integrins (Figs. 1*A* and 4), suggesting a generalizable mechanism. A remarkable difference between β_3_ and α_IIb_ TM domains is that most of the tested α_IIb_ TM proline mutations increased α_IIb_β_3_ inside-out activation except one inhibitory proline mutation, α_IIb_-I982P present in the C-terminal portion of α_IIb_ TM α-helix, suggesting that the α_IIb_ TM domain is less tolerant to the α-helix disrupting proline mutations than β_3_ TM domain. These data demonstrate that a continuous α-helical structure is required for both α and β integrin TM domains to maintain a normal function of integrin inside-out activation.

The inhibitory β_3_-A711P mutation was previously identified by random mutagenesis (15). Structural studies by NMR demonstrated a kink conformation induced by β_3_-A711P in the isolated β_3_ TM fragment and in complex with α_IIb_ TM domain (15, 36). The structural and thermodynamic analysis suggests that the β_3_-A711P mutation stabilizes the α_IIb_β_3_ TM association probably due to the kink-mediated repacking of α_IIb_β_3_ TM heterodimer (36). Besides β_3_-A711P, we identified three more β_3_ TM proline mutations, β_3_-L698P, β_3_-L705P, and β_3_-I707P, all of which reduce the inside-out activation of α_IIb_β_3_. We also found that these proline mutations have different levels of inhibitory effect, which is similar to the effect of activating mutations. Similar to β_3_-A711P, these inhibitory proline mutations are likely to stabilize the α_IIb_β_3_ TM association by altering the packing of β_3_ TM α-helix on α_IIb_. The stabilized α_IIb_β_3_ TM interaction then increases the energy barrier for activation. This is consistent with our data showing that the α_IIb_β_3_ activation in the presence of β_3_-L705P-A711P mutant requires the highly activating mutation α_IIb_-F992A-F993A (Fig. 2*C*).

The physiological inside-out activation of integrin is initiated by the binding of talin head domain to the β CT, which disrupts the α-β TM association leading to conformational activation of ectodomain (37). It was suggested that the binding of talin head may change the topology of β_3_ TM domain, as determined using the isolated β_3_ TM-CT fragment in model membranes (35). We found that the β_3_-L705P and -A711P mutations completely abolished talin1-head-induced α_IIb_β_3_ activation. Given that the proline-mediated kink formation disrupts the integrity and rigidity of β_3_ TM α-helix, it may interrupt the talin1-head-induced topology change of β_3_ TM domain. We tested this possibility by introducing a flexible loop insert at the intracellular boundary of β_3_ TM domain, where it does not affect talin1 head binding but interrupts the rigid α-helical structure. These α-helix disruption mutations also dramatically reduced talin1-head-induced α_IIb_β_3_ activation, suggesting that a rigid α-helical structure is required for talin head-induced conformational change of β_3_ TM domain.

Another key observation in our study is that the inhibitory β_3_ TM proline mutations block α_IIb_β_3_-mediated cell spreading, a hallmark of integrin outside-in signaling. Our previous studies using disulfide cross-linking demonstrated that the separation or conformational change of α_IIb_β_3_ TM domain is required for integrin outside-in signaling (38). Given that the proline mutations stabilize the TM association as discussed above, the extracellular ligand-induced separation or conformational change of the TM domain may be inhibited by the stabilizing TM proline mutations. However, the stabilizing α_IIb_-I982P mutation exerted no effect on cell spreading. These data suggest that the integrity of β_3_ TM α-helical structure is critical for the outside-in signal transmission. During outside-in signaling, integrin transmits both chemical and mechanical signals across the cell membrane (34). The mechanical stress generated by the binding of extracellular ligands to the ectodomain and cytoskeleton molecules to the β CT exerts tensile force to the TM domain. Our data suggest that a continuous and rigid α-helical structure of β_3_ TM domain is critical for the sensing and regulation of molecular tension.

The structures of α_IIb_β_3_ TM-CT domains have been studied as isolated fragments in the model membrane or detergent micelles (10, 12, 13, 19, 20). However, complexed results were obtained about the TM topology within the membrane and the potential structural changes upon integrin activation. This is possibly due to the absence of integrin extracellular domains and the nonnative membrane environment in those studies. An elegant study using a cysteine scanning accessibility method investigated the topology of intracellular borders of both α_IIb_ and β_3_ TM domains in the context of full-length integrin on the cell membrane (17). Using the same approach, we analyzed both the extracellular and intracellular membrane interfaces of α_IIb_β_3_ TM domains in the resting and active conditions. In our study, we used both membrane-permeable and -impermeable biotin-maleimide and performed the labeling on ice or at 4 °C to minimize the effect of membrane thermodynamics. Also, we used 2-BP to inhibit the potential intracellular cysteine palmitoylation that is known to block the -SH group for maleimide labeling. This enables us to detect the BM labeling of the intracellular cysteine mutations such as β_3_ D723C, R724C, K725C, and E726C, which were not detected in the previous study when 2-BP was not used (17). Our study allows us to define both the outer and inner membrane boundaries of α_IIb_β_3_. For β_3_ integrin, the membrane-embedded portion is I693-H722, which is seven residues longer than the predicted 23-residue TM domain. Given the continuous α-helical structure of this 30-residue fragment, it requires a tilt of β_3_ TM domain to match the thickness of lipid bilayer (Fig. 1*E*). This is in general consistent with the model suggested based on structural studies of α_IIb_β_3_ TM heterodimer by NMR in lipid bicelles (13).

Our BM labeling results suggest the membrane-embedded portion for α_IIb_ is W968-R995, showing that the conserved GFFKR motif is masked by a membrane except for K994. This is consistent with a reverse turn conformation of GFFKR as determined by structural studies (11, 13), which allows the GFF and R to be buried while exposing K994 (Fig. 1*E*). However, due to the activating effect of GFFKR cysteine substitutions, it was suggested by the previous study that the BM labeling pattern of the GFFKR motif reflects an active conformation (17). To compensate for the activating effect of GFFKR cysteine mutations, we introduced an interchain disulfide bridge at the extracellular membrane-proximal portion to restore α_IIb_β_3_ to the resting state by stabilizing TM association. This activation-reversing mutation did not change the pattern of BM labeling of the GFFKR motif, suggesting that the GFFKR motif is membrane embedded in the resting state. In contrast to the β_3_ TM domain, the reverse turn conformation of the GFFKR motif allows the αIIb TM α-helix to adopt a perpendicular orientation in the lipid bilayer (Fig. 1*E*).

The membrane tilt of β3 TM domain has been suggested to play a role in α_IIb_β_3_ inside-out activation (15, 35). How the TM tilt is determined is still a debate (18). NMR studies of the monomeric β_3_ TM-CT fragment with and without β_3_-K716E mutation revealed a change of membrane embedding, leading to the conclusion that the β_3_ TM tilt is determined by the snorkeling of β_3_-K716 side chain to form a lysine/phospholipid ion pair (15). However, the observed change of membrane embedding in the presence of β_3_-K716E was demonstrated to be an experimental artifact by another NMR study (18), questioning the β_3_-K716 snorkeling model in defining β_3_ TM tilt. Using a monomeric β_3_-tail-TMCT construct composed of β_3_ β-tail and TM-CT domains expressed on the cell surface without α_IIb_, our BM labeling experiments revealed a different membrane embedding compared with α_IIb_β_3_ heterodimer, which shows an increased exposure of both extracellular and intracellular borders, indicating the change of TM tilt. This data suggests that the β_3_ TM tilt is very likely to be determined by the association with α_IIb_ TM domain. Structural analysis of α_IIb_β_3_ TM heterodimer reveals two close contacts within cell membrane, one formed by the α_IIb_ GXXXG motif, the other formed by the α_IIb_ GFFKR (Fig. 1*E*). The β_3_-K716 side chain hydrogen bonding with the backbone oxygens of α_IIb_ GFFKR motif (11), along with the close packing at the α_IIb_ GXXXG motif favors the tilt topology of β_3_ TM domain (Fig. 1*E*). The activation inhibitory proline mutations may further stabilize the β_3_ TM tilt and α_IIb_β_3_ TM association. A perpendicular and rigid α-helical structure of α_IIb_ TM domain is critical in maintaining the resting state of α_IIb_β_3_ since our mutagenesis study shows that α_IIb_ is much less tolerant to TM proline mutations than β_3_.

Current model suggests that α_IIb_β_3_ activation involves a disruption of α-β TM association, which may alter the TM topology of either α_IIb_, or β_3_, or both (4, 17). The TM association can be disturbed by many modulations including disruptive mutations, talin and kindlin binding, and even membrane tension (39), which all lead to integrin activation. Unexpectedly, our BM labeling experiments failed to detect any significant topology changes upon soluble ligand binding or overexpression of talin1 head domain. A previous study using the same approach also did not show the change of BM labeling for the intracellular portion of β_3_ TM domain in the presence of the activating α_IIb_-F992A-F993A mutation (17). A possible reason is that the BM labeling approach is not sensitive enough to detect the subtle and/or transient topology changes of the TM domain. The change of membrane embedding depends on the type of movement of TM α-helix relative to the lipid bilayer. A tilt or piston-like movement can lead to the change of membrane embedding, while a small rotation may not affect the embedding. All of these structural changes are expected to disrupt α-β TM interaction, leading to integrin activation. A study using membrane-embedded monomeric β3 TM-CT fragment bearing environment-sensitive fluorophores showed an increase of membrane embedding upon talin1 head binding (35). However, our BM labeling of monomeric β_3_-tail-TMCT shows a change of membrane embedding even without talin head, suggesting that the β_3_ TM domain adopts a different topology in the absence of α_IIb_ TM domain. The observation of talin-induced topology change of monomeric β_3_ TMCT peptide in the absence of α_IIb_ may not represent the physiological condition. Nonetheless, the topology changes may happen when tension applied to the β_3_ TMCT during cell adhesion and spreading as shown by our current study. A topology change of α_IIb_ TMCT may also contribute to α_IIb_β_3_ activation as suggested by the previous study using BM labeling (17). The membrane distal region of α_IIb_ CT may be required for the topology change (14). A topology change of α_IIb_ TM α-helical structure is more prone to disturb the α-β TM interaction according to our proline mutagenesis study. Alerting the α_IIb_ TM structure may require a lower energy cost than changing the β_3_ TM tilt. A subtle rotation of TM α-helix will be more efficient to disturb TM association than changing the membrane embedding. Unfortunately, this type of TM structural change cannot be detected by BM labeling. Despite the substantial efforts on α_IIb_β_3_ TM studies, more questions remain to be answered using innovative approaches.

## Experimental procedures

### DNA constructs and mutagenesis

The plasmids for α_IIb_, β_3_, α_L_, α_5_, and β_1_ integrins were as described before (14, 40, 41, 42). The DNA construct of EGFP-tagged mouse talin1-head domain was as described (43). The β_3_-tail-TMCT construct composed of β_3_ β-tail, TM, and CT domains (residues P605-T762) was cloned into a modified pIRES2-EGFP vector with an N-terminal signal peptide derived from murine IgG kappa V followed by a protein C epitope tag. Mutations were made using site-directed mutagenesis with the QuikChange kit (Agilent Technologies). All the introduced mutations were confirmed by DNA sequencing.

### Antibodies and ligands

PAC-1 (BD Bioscience) is a ligand-mimetic mAb (IgM) for the activated α_IIb_β_3_ integrin (44). AP3 is a conformation-independent anti-β_3_ mAb (45) and was conjugated with Alexa Fluor 488 (Thermo Fisher Scientific). 10E5 is an anti-α_IIb_β_3_ complex specific mAb (31, 46). 314.5 is an anti-α_IIb_ mouse mAb that binds to calf-2 domain. H-96 is a rabbit anti-β_3_ polyclonal IgG (Santa Cruz Biotechnology). H-160 is a rabbit anti-α_IIb_ polyclonal IgG (Santa Cruz Biotechnology). PE-labeled MAR4 (BD Bioscience) is a nonfunctional anti-β_1_ mAb. PE-labeled TS2/4 (BioLegend) is a nonfunctional anti-α_L_ mAb (47). KIM127 that binds to I-EGF-2 domain and mAb 24 (m24) that binds to βI domain are anti-β_2_ conformation-dependent mAbs (28, 29, 48, 49). Rabbit anti-protein C tag was from GenScript. Anti-protein C matrix beads were from Sigma-Aldrich. IRDye 800-labeled streptavidin and IRDye 680-labeled goat anti-rabbit (or mouse) IgG were from LI-COR Biosciences. Human fibrinogen (Plasminogen, von Willebrand Factor, and Fibronectin Depleted) was from Enzyme Research Laboratories. Human fibronectin and human ICAM-1 with a C-terminal Fc tag of human IgG1 (ICAM-1-Fc) were from Sigma-Aldrich and Sino-Biological, respectively.

### Ligand and antibody-binding assay

The detailed protocol of ligand and antibody-binding assay for integrin was as we published before (50). HEK293FT cells were used for the transient transfection of α_IIb_β_3_ and α_L_β_2_ integrins. HEK293FT- α_5_β_1_-KO cells were used for α_5_β_1_ integrin (51). The ligand binding to integrin-transfected cells was performed in HBSGB buffer (25 mM HEPES, pH 7.4, 150 mM NaCl, 5.5 mM glucose, and 1% BSA) plus 1 mM CaCl_2_ and 1 mM MgCl_2_ (Ca/Mg). The KIM127 and m24 binding were performed in HBSGB plus Ca/Mg or 0.2 mM CaCl_2_ and 2 mM MnCl_2_ (Ca/Mn). The ligand or conformation-dependent mAb binding was presented as normalized mean florescence intensity (MFI), *i.e.*, the ligand or mAb MFI as a percentage of integrin MFI measured by AP3 for α_IIb_β_3_, TS2/4 for α_L_β_2_, and MAR4 for α_5_β_1_. When EGFP-TH was coexpressed with integrin constructs, the EGFP and integrin double-positive cells were gated for MFI calculation. The expression of EGFP-TH was measured based on the MFI of EGFP.

### Cell adhesion and spreading assay

HEK293 cells were used to generate stable transfections of wild type and proline mutants of α_IIb_β_3_. The single cell clones that have comparable surface expression of α_IIb_β_3_ were selected for cell adhesion and spreading assay as described before (38, 40). In brief, the Delta T dish (Bioptechs) was coated with 5 μg/ml PAC-1 or 25 μg/ml fibrinogen in PBS buffer at 4 °C overnight and then blocked with 1% BSA at 37 °C for 1 h. The cells in suspension were washed once with DMEM without serum and seeded onto the Delta T dish at 37 °C for 1 h. The attached cells were washed with DMEM and fixed with 3.7% formaldehyde in PBS at 25 °C for 5 min. The fixed cells were first immunostained with mAb AP3 and then permeabilized with 0.05% Triton X-100 in PBS, followed by staining with Alexa Fluor 546 labeled phalloidin (Thermo Fisher Scientific) and DAPI. Cells were imaged with EVOS digital inverted fluorescence microscope. The cell areas of 40 to 50 total cells for each independent experiment were measured using ImageJ and averaged.

### Biotin maleimide labeling and immunoprecipitation

The cell-membrane-permeable biotin-maleimide was from Sigma-Aldrich. The cell-impermeant maleimide-PEG11-biotin was from Thermo Fisher Scientific. The concentrations of biotin-maleimide and maleimide-PEG11-biotin and the labeling time were optimized using the well-exposed and well-embedded cysteine mutants. The labeling temperature was also compared between 25 °C and 0 °C (or 4 °C). We also compared the maleimide-PEG11-biotin and biotin-maleimide for labeling the cysteine mutations at the extracellular portion of the TM domain in intact cells, or the cysteine mutations at the intracellular portion of the TM domain after physically breaking the cell membrane. No obvious differences were found among the different conditions. We used maleimide-PEG11-biotin for labeling the extracellular TM cysteine mutations and biotin-maleimide for the intracellular TM-CT cysteine mutations. The labeling was performed following the published protocol with some modifications (17). HEK293FT cells cultured in a 12-well plate were transfected with integrin constructs. For the transfection of intracellular cysteine mutants, the cells were pretreated with 15 μg/ml 2-bromopalmitate (Sigma-Aldrich) to inhibit the potential cysteine palmitoylation (11). The transfected cells were suspended and washed with 0.5 ml PBSCM (140 mM NaCl, 3 mM KCl, 6.5 mM Na2HPO4, 1.5 mM KH2PO4, pH 7.0 plus 1 mM CaCl2, and 1 mM MgCl2). The cells suspended in 0.5 ml PBSCM were labeled with 0.2 mM biotin-maleimide or 0.1 mM maleimide-PEG11-biotin on ice for 30 min and then stopped by adding 1 mM glutathione and washed once with 0.5 ml PBSCM. The cells were lysed in 0.5 ml IPB buffer (20 mM Tris-HCl, pH 7.5, 150 mM NaCl, 1% Triton X-100), 0.5% sodium deoxycholate, plus 1 mM CaCl2, and 1 mM MgCl2 containing 0.2% BSA and protease inhibitors cocktail (Roche Applied Science) on ice for 10 min. The cell lysates were cleared by centrifugation at 15,000 rpm for 15 min. The proteins were immunoprecipitated for 3 h at 4 °C by mAb 10E5 and protein A agarose beads for α_IIb_β_3_ or anti-PC matrix beads for β_3_-tail-TMCT.

For the labeling in the presence of the RGD-mimetic ligand eptifibatide, the cells were incubated with 20 μM eptifibatide at 25 °C for 15 min and then labeled on ice for 30 min. For the labeling in the presence of EGFP-TH, the cells were cotransfected with α_IIb_β_3_ plus EGFP-TH or EGFP. For the labeling of spreading cells, the cells suspended in DMEM without serum were allowed to spread on the plate coated with 50 μg/ml fibrinogen at 37 °C for 1 h and then washed with PBSCM and labeled on-site at 4 °C for 30 min.

### SDS-PAGE and immunoblotting

The immunoprecipitated proteins were resolved on 7.5% or 12% SDS–polyacrylamide gels under reducing or nonreducing conditions and transferred to PVDF membranes. The membranes were blocked with Intercept (PBS) blocking buffer (LI-COR Biosciences) at 25 °C for 1 h and then incubated for 1 h at 25 °C with 1 μg/ml each of 314.5 (for α_IIb_), H-160 (for α_IIb_), H-96 (for β_3_), AP3 (for β_3_ under nonreducing condition), or anti-PC (for β_3_-tail-TMCT) diluted in Intercept (PBS) blocking buffer plus 0.1% SDS and 0.1% Tween 20. The membranes were washed once with 10 ml TBS-T buffer for 15 min and then washed three times with TBS-T buffer for 5 min each and then incubated at 25 °C for 1 h with IRDye 800 streptavidin plus IRDye 680 goat anti-rabbit (or mouse) IgG diluted in Intercept (PBS) blocking buffer plus 0.1% SDS and 0.1% Tween 20. The membranes were washed and scanned with the Odyssey Infrared Imaging System (LI-COR Biosciences).

### Statistical analysis

Statistical analysis was carried out on at least three individual datasets and analyzed with GraphPad Prism software. Unpaired two-tailed *t* test was performed between control and treated experimental groups. *p*-values ≤0.05 were considered significant.

## Data and materials availability

All data are available in the main text.

## Conflict of interest

Authors declare that they have no competing interests.
